# Supplementary material for: Linking traits based on their shared molecular mechanisms
Source: eLife. 2015 Mar 17;4:e04346. doi: 10.7554/eLife.04346 (PMC4362207; doi:10.7554/eLife.04346)
Supplement: Supplementary file 1. — (A) Traits in the GEMOT modules of the BXD mouse strains. Shown is a module identifier (column 1) and details about a trait within it, including the trait title (column 2), PubMed record of the publication from which it is taken (column 3), the trait index in the GeneNetwork database (Wang et al., 2003; columns 4), and a short title of the trait as indicated in Figure 3B (column 5). (B) Drivers in the GEMOT modules of the BXD mouse strains. Shown is a module identifier (column 1), and details about a driver within it, including the gene symbol (column 2), its entrez identifier (column 3), genomic position (column 4), the type of association in the module (column 5), and its gene causality p value score (column 6). (C) Representative variants for causality testing in the tripartite modules of the BXD mouse strains. Shown is a tripartite module identifier and its GEMOT module identifier, if relevant (columns 1 and 2, respectively), the genomic position of the module's genomic interval (column 3), and the name and position of the representative variant (columns 4 and 5, respectively). DOI: http://dx.doi.org/10.7554/eLife.04346.027 [file elife04346s001.doc]

# Supplementary File 1A

| **Module Identifier** | **Trait title** | **PubMed Identifier** | **Gene-Network Identifier** | **Short title** |
| --- | --- | --- | --- | --- |
| 1 | Anxiety assay + ethanol treated (zero maze), 5 minutes, for males and females | 18830130 | 12450 | Ethanol response, anxiety assay , 5 min |
| 1 | Anxiety assay + ethanol treated (zero maze), 10 minutes, for males and females | 18830130 | 12451 | Ethanol response, anxiety assay , 10 min |
| 2 | Place preference, 90-105 min after morphine injection (50 mg/kg ip) for males | 19958391 | 11312 | Morphine place preference , 90-105 min |
| 2 | Place preference 105-120 min after morphine injection (50 mg/kg ip) for males | 19958391 | 11313 | Morphine place preference , 105-120 min |
| 2 | Distance (cm) travelled, 90-105 min after morphine injection (50 mg/kg ip) for males | 19958391 | 11323 | Morphine distance travelled , 90-105 min |
| 2 | Distance (cm) travelled 105-120 min after morphine injection (50 mg/kg ip) for males | 19958391 | 11324 | Morphine distance travelled , 105-120 min |
| 2 | Distance (cm) travelled 120-135 min after morphine injection (50 mg/kg ip) for males | 19958391 | 11325 | Morphine distance travelled , 120-135 min |
| 2 | Distance (cm) travelled 75-90 min after morphine injection (50 mg/kg ip) for males | 19958391 | 11333 | Morphine distance travelled , 75-90 min |
| 2 | Place preference 0-180 min after morphine injection (50 mg/kg ip) for males (n beam breaks) | 19958391 | 11337 | Morphine place preference , 0-180 min |
| 2 | Distance (cm) travelled 0-180 min after morphine injection (50 mg/kg ip) for males | 19958391 | 11338 | Morphine distance travelled , 0-180 min |
| 2 | Vertical activiy, 0-180 min after morphine injection (50 mg/kg ip) for males (n beam breaks) | 19958391 | 11340 | Morphine vertical activiy , 0-180 min |
| 2 | Vertical activiy, 90-105 min after morphine injection (50 mg/kg ip) for males (n beam breaks) | 19958391 | 11364 | Morphine vertical activiy , 90-105 min |
| 2 | Vertical activiy, 105-120 min after morphine injection (50 mg/kg ip) for males (n beam breaks) | 19958391 | 11365 | Morphine vertical activiy , 105-120 min |
| 2 | Vertical activiy, 120-135 min after morphine injection (50 mg/kg ip) for males (n beam breaks) | 19958391 | 11366 | Morphine vertical activiy , 120-135 min |
| 2 | Vertical activiy, 75-90 min after morphine injection (50 mg/kg ip) for males (n beam breaks) | 19958391 | 11374 | Morphine vertical activiy , 75-90 min |
| 2 | Distance (cm) travelled 90-105 min after morphine injection (50 mg/kg ip) for males and females | 19958391 | 11837 | Morphine distance travelled , 90-105 min |
| 2 | Distance (cm) travelled 105-120 min after morphine injection (50 mg/kg ip) for males and females | 19958391 | 11838 | Morphine distance travelled , 105-120 min |
| 2 | Distance (cm) travelled 0-180 min after morphine injection (50 mg/kg ip) for males and females | 19958391 | 11852 | Morphine distance travelled , 0-180 min |
| 2 | Vertical activiy, 0-15 min after morphine injection (50 mg/kg ip) for males and females (n beam breaks) | 19958391 | 11881 | Morphine vertical activiy , 0-15 min |
| 3 | Ethanol response (2 mg/kg ip), conditioned place preference (CPP) for the ethanol-paired grid compartment in experimental group males [s/min] | 7480533 | 10097 | Ethanol response, place preference |
| 3 | Eye, morphology, visual system: Eye weight corrected for age, sex, body and brain weight [mg] | 10102277 | 10667 | Eye weight |
| 3 | Eye, morphology, visual system: Lens weight [mg] | 10102277 | 10668 | Lens weight |

# Supplementary File 1A - cont.

| 3 | Eye, morphology, visual system: Eye weight, regression corrected for age, sex, body and brain weight [mg] | 10102277 | 10671 | Eye weight |
| --- | --- | --- | --- | --- |
| 3 | Pain response, thermal nociception, Hargreaves' test for males [units] | 19958391 | 11307 | Thermal nociception, Hargreaves' test |
| 4 | Cancer: Hepatocellular carcinoma tumor multiplicities induced by N,N-diethylnitrosamine (DEN 0.01 ml/g, ip at postnatal day 12), mean tumor multiplicity in liver at 32 weeks in male [n] | 7705639 | 10370 | Tumor multipliciy in liver |
| 4 | Central nervous system, morphology, aging: Polyglucosan bodies in 18-month-old females (also see AXB/BXA data) [log number] | 11113616 | 10686 | Hippocampus age-related lesions |
| 4 | Central nervous system, morphology: Hippocampus, dorsal hippocampus volume, age-adjusted residuals [mm^3] | 17081266 | 10755 | Dorsal hippocampus volume |
| 4 | Hematopoietic stem cell number (cobblestone-area-forming cells per femur at 35 days of age from 3 females) | 17220891 | 10890 | HSC number |
| 4 | Cocaine response, locomotion (cm) 0-15 min for females | 19958391 | 11779 | Cocaine response, locomotion , 0-15 min |
| 5 | Pain response, thermal nociception, hot-plate test for males | 9315917 | 10426 | Thermal nociception, hot-plate test |
| 5 | Ethanol response [4/g/kg ip], plasma corticosterone concentration 6 hr after injection in males [ug/dl] | 8748968 | 10580 | Ethanol response, plasma corticosterone concentration |
| 5 | Anxiety assay (plus maze, open arms) for males | 19958391 | 11455 | Anxiety assay, open arms zero maze |
| 5 | Anxiety assay (plus maze, closed arms) for males | 19958391 | 11456 | Anxiety assay, closed arms zero maze |
| 5 | Place preference 120-135 min after morphine injection (50 mg/kg ip) for females | 19958391 | 11571 | Morphine place preference , 120-135 min |
| 5 | Distance (cm) travelled 120-135 min after morphine injection (50 mg/kg ip) for females | 19958391 | 11582 | Morphine distance travelled , 120-135 min |
| 6 | Place preference 30-45 min after morphine injection (50 mg/kg ip) for males | 19958391 | 11319 | Morphine place preference , 30-45 min |
| 6 | Place preference,15-30 min after morphine injection (50 mg/kg ip) for males and females | 19958391 | 11832 | Morphine place preference , 15-30 min |
| 7 | Place preference, 90-105 min after morphine injection (50 mg/kg ip) for males | 19958391 | 11312 | Morphine place preference , 90-105 min |
| 7 | Place preference 60-75 min after morphine injection (50 mg/kg ip) for males | 19958391 | 11321 | Morphine place preference , 60-75 min |
| 7 | Place preference 75-90 min after morphine injection (50 mg/kg ip) for males | 19958391 | 11322 | Morphine place preference , 75-90 min |
| 7 | Distance (cm) travelled, 90-105 min after morphine injection (50 mg/kg ip) for males | 19958391 | 11323 | Morphine distance travelled , 90-105 min |
| 7 | Distance (cm) travelled 60-75 min after morphine injection (50 mg/kg ip) for males | 19958391 | 11332 | Morphine distance travelled , 60-75 min |
| 7 | Distance (cm) travelled 75-90 min after morphine injection (50 mg/kg ip) for males | 19958391 | 11333 | Morphine distance travelled , 75-90 min |
| 7 | Place preference 0-180 min after morphine injection (50 mg/kg ip) for males (n beam breaks) | 19958391 | 11337 | Morphine place preference , 0-180 min |
| 7 | Distance (cm) travelled 0-180 min after morphine injection (50 mg/kg ip) for males | 19958391 | 11338 | Morphine distance travelled , 0-180 min |
| 7 | Vertical activiy, 45-60 min after morphine injection (50 mg/kg ip) for males (n beam breaks) | 19958391 | 11372 | Morphine vertical activiy , 45-60 min |

# Supplementary File 1A - cont.

| 7 | Place preference,105-120 min after morphine injection (50 mg/kg ip) for males and females | 19958391 | 11827 | Morphine place preference , 105-120 min |
| --- | --- | --- | --- | --- |
| 7 | Place preference,75-90 min after morphine injection (50 mg/kg ip) for males and females | 19958391 | 11836 | Morphine place preference , 75-90 min |
| 7 | Distance (cm) travelled 105-120 min after morphine injection (50 mg/kg ip) for males and females | 19958391 | 11838 | Morphine distance travelled , 105-120 min |
| 7 | Distance (cm) travelled 60-75 min after morphine injection (50 mg/kg ip) for males and females | 19958391 | 11846 | Morphine distance travelled , 60-75 min |
| 7 | Place preference,0-180 min after morphine injection (50 mg/kg ip) for males and females | 19958391 | 11851 | Morphine place preference , 0-180 min |
| 7 | Vertical activiy, 45-60 min after morphine injection (50 mg/kg ip) for males and females (n beam breaks) | 19958391 | 11886 | Morphine vertical activiy , 45-60 min |
| 7 | Vertical activiy, 60-75 min after morphine injection (50 mg/kg ip) for males and females (n beam breaks) | 19958391 | 11887 | Morphine vertical activiy , 60-75 min |
| 7 | Vertical activiy, 75-90 min after morphine injection (50 mg/kg ip) for males and females (n beam breaks) | 19958391 | 11888 | Morphine vertical activiy , 75-90 min |
| 8 | Central nervous system, morphology: Cerebellum internal granule layer (IGL) volume without adjustment [mm^3] | 11438585 | 10006 | Cerebellum IGL volume |
| 8 | Endocrinology, neuroendocrine system, morphology: Adrenal zona glomerulosa width for males [um] | 21347846 | 11268 | Adrenal gland size |
| 8 | Distance (cm) travelled 15-30 min after morphine injection (50 mg/kg ip) for males | 19958391 | 11329 | Morphine distance travelled , 15-30 min |
| 8 | Distance (cm) travelled 60-75 min after morphine injection (50 mg/kg ip) for males | 19958391 | 11332 | Morphine distance travelled , 60-75 min |
| 8 | Vertical activiy, 30-45 min after morphine injection (50 mg/kg ip) for males (n beam breaks) | 19958391 | 11371 | Morphine vertical activiy , 30-45 min |
| 8 | Vertical activiy, 45-60 min after morphine injection (50 mg/kg ip) for males (n beam breaks) | 19958391 | 11372 | Morphine vertical activiy , 45-60 min |
| 8 | Place preference 75-90 min after morphine injection (50 mg/kg ip) for females | 19958391 | 11579 | Morphine place preference , 75-90 min |
| 8 | Distance (cm) travelled 75-90 min after morphine injection (50 mg/kg ip) for females | 19958391 | 11590 | Morphine distance travelled , 75-90 min |
| 8 | Place preference,60-75 min after morphine injection (50 mg/kg ip) for males and females | 19958391 | 11835 | Morphine place preference , 60-75 min |
| 8 | Distance (cm) travelled 60-75 min after morphine injection (50 mg/kg ip) for males and females | 19958391 | 11846 | Morphine distance travelled , 60-75 min |
| 8 | Vertical activiy, 0-180 min after morphine injection (50 mg/kg ip) for males and females (n beam breaks) | 19958391 | 11854 | Morphine vertical activiy , 0-180 min |
| 8 | Vertical activiy, 30-45 min after morphine injection (50 mg/kg ip) for males and females (n beam breaks) | 19958391 | 11885 | Morphine vertical activiy , 30-45 min |
| 8 | Vertical activiy, 45-60 min after morphine injection (50 mg/kg ip) for males and females (n beam breaks) | 19958391 | 11886 | Morphine vertical activiy , 45-60 min |
| 8 | Vertical activiy, 60-75 min after morphine injection (50 mg/kg ip) for males and females (n beam breaks) | 19958391 | 11887 | Morphine vertical activiy , 60-75 min |
| 8 | Vertical activiy, 75-90 min after morphine injection (50 mg/kg ip) for males and females (n beam breaks) | 19958391 | 11888 | Morphine vertical activiy , 75-90 min |

# Supplementary File 1B

| **Module Identifier** | **Gene symbol** | **entrez Identifier** | **Genomic position** | ***Cis/Trans*** | **Gene causality score** |
| --- | --- | --- | --- | --- | --- |
| 1 | Slc37a4 | 14385 | chr9:44206259-44211049 | *Trans* | 4.30E-03 |
| 2 | Ggt1 | 14598 | chr10:75024349-75048945 | *Trans* | 3.33E-16 |
| 2 | Lrrk2 | 66725 | chr15:91503606-91646551 | *Trans* | 5.90E-14 |
| 2 | Rexo2 | 104444 | chr9:48276619-48288716 | *Trans* | 4.86E-14 |
| 2 | Ntng2 | 171171 | chr2:29050061-29108525 | *Trans* | 8.16E-14 |
| 2 | p35 | 12569 | chr11:80290525-80294686 | *Trans* | 1.38E-12 |
| 2 | Cish | 12700 | chr9:107199020-107204292 | *Trans* | 1.99E-11 |
| 2 | Klf7 | 93691 | chr1:64076021-64168856 | *Cis* | 1.62E-09 |
| 2 | Ibtk | 108837 | chr9:85580963-85637760 | *Trans* | 1.55E-08 |
| 2 | Soga1 | 320706 | chr2:156841535-156904990 | *Trans* | 6.10E-09 |
| 2 | Ctnnal1 | 54366 | chr4:56823807-56878060 | *Trans* | 1.25E-08 |
| 2 | Map7d3 | 320923 | chr20:54051035-54075503 | *Trans* | 3.20E-08 |
| 2 | Mmp13 | 17386 | chr9:7272545-7283331 | *Trans* | 1.81E-07 |
| 2 | Ube2h | 22214 | chr1:59157437-59176940 | *Trans* | 2.05E-07 |
| 2 | Tmem237 | 381259 | chr1:59157437-59176940 | *Trans* | 7.03E-07 |
| 2 | Nop10 | 66181 | chr2:112102083-112103426 | *Trans* | 1.81E-06 |
| 2 | Ankrd13d | 68423 | chr19:4270182-4283137 | *Trans* | 3.95E-06 |
| 2 | Slc22a20 | 381203 | chr19:5970234-5986143 | *Trans* | 9.63E-06 |
| 2 | Idh1 | 15926 | chr1:65205190-65225755 | *Cis* | 3.10E-05 |
| 2 | Scamp1 | 107767 | chr13:94971265-95055812 | *Trans* | 1.34E-05 |
| 2 | Dclk2 | 70762 | chr3:86590081-86724774 | *Trans* | 1.89E-05 |
| 2 | Tonsl | 72749 | chr15:76456668-76470341 | *Trans* | 3.59E-04 |
| 2 | Qsox1 | 104009 | chr1:157625285-157660013 | *Trans* | 3.17E-05 |
| 2 | Chi3l1 | 12654 | chr1:136078753-136086758 | *Trans* | 3.42E-05 |
| 2 | Armc3 | 70882 | chr2:19120929-19231868 | *Trans* | 6.29E-05 |
| 2 | Ppp1r15b | 108954 | chr1:135027720-135036360 | *Trans* | 1.35E-04 |
| 2 | Lrg1 | 76905 | chr17:56259103-56261369 | *Trans* | 1.32E-04 |
| 2 | Rai14 | 75646 | chr15:10498734-10643295 | *Trans* | 2.12E-03 |
| 2 | Asb7 | 117589 | chr7:73789451-73834482 | *Trans* | 5.86E-04 |
| 2 | Plscr3 | 70310 | chr11:69659878-69664545 | *Trans* | 8.36E-04 |
| 2 | Noc3l | 57753 | chr19:38863621-38893690 | *Trans* | 1.54E-03 |
| 2 | Eef1e1 | 66143 | chr13:38737563-38750879 | *Trans* | 1.53E-03 |
| 2 | Tdrkh | 72634 | chr3:94217240-94235413 | *Trans* | 1.51E-03 |
| 3 | Sytl1 | 269589 | chr4:132809005-132819028 | *Trans* | 2.89E-05 |
| 3 | Cd52 | 23833 | chr4:133638363-133650997 | *Cis* | 2.33E-03 |
| 3 | Eya3 | 14050 | chr4:132194902-132280680 | *Trans* | 4.43E-03 |
| 4 | Ythdf2 | 213541 | chr4:132184912-132212303 | *Trans* | 1.07E-06 |
| 4 | Aldh6a1 | 104776 | chr12:85772801-85791953 | *Trans* | 7.85E-06 |
| 4 | Sypl | 19027 | chr12:33638756-33663915 | *Trans* | 3.88E-05 |
| 4 | Pced1a | 319513 | chr2:130242983-130250437 | *Trans* | 8.22E-05 |
| 4 | Dyt1 | 30931 | chr2:30816147-30823453 | *Trans* | 6.23E-05 |
| 4 | Nsun5 | 100609 | chr5:135845823-135852675 | *Trans* | 9.37E-05 |
| 5 | RinL | 320435 | chr7:28788969-28798963 | *Trans* | 5.92E-05 |
| 6 | Ndufaf7 | 73694 | chr17:79336476-79346839 | *Trans* | 1.90E-03 |
| 6 | Myo1d | 338367 | chr11:80295628-80593527 | *Trans* | 3.53E-03 |
| 6 | Fbxo30 | 71865 | chr10:11001128-11017850 | *Trans* | 2.73E-03 |

Supplementary File 1B - cont.

| 7 | Dexi | 58239 | chr16:10530300-10543147 | *Trans* | 1.30E-09 |
| --- | --- | --- | --- | --- | --- |
| 7 | Pten | 19211 | chr19:32831987-32900650 | *Trans* | 4.58E-03 |
| 7 | Clec16a | 74374 | chr16:10545457-10744971 | *Trans* | 4.15E-05 |
| 7 | Mpg | 268395 | chr11:32126505-32132700 | *Trans* | 7.38E-07 |
| 8 | Lpp | 210126 | chr16:24393576-24981116 | *Trans* | 2.83E-03 |
| 8 | Matk | 17179 | chr10:80715680-80726110 | *Trans* | 5.32E-09 |
| 8 | Sbno2 | 216161 | chr10:79519765-79565447 | *Trans* | 7.33E-06 |

Supplementary File 1C

| **Tripartite Module identifier** | **GEMOT Module identifier** | **Genomic interval** | **Representative variant** | **Representative locus** |
| --- | --- | --- | --- | --- |
| 1 | 1 | chr1:29231426-42216522 | rs13475810 | chr1:36052584 |
| 2 | 2 | chr1:62750756-68809667 | rs13475891 | chr1:62750756 |
| 3 | 3 | chr4:133563658-142772319 | rs3023025 | chr4:142772319 |
| 4 | 4 | chr11:8736728-19472433 | rs3714397 | chr11:8736728 |
| 5 | 5 | chr14:59434131-69379833 | rs13482218 | chr14:63105548 |
| 6 | 6 | chr15:12602461-44717039 | rs13482436 | chr15:12602461 |
| 7 | 7 | chr16:10701009-15051845 | rs4161101 | chr16:10701009 |
| 8 | 8 | chr19:48744594-56457053 | CEL-19_48014568 | chr19:48744594 |
| 9 | - | chr4:81604581-95416080 | rs3663299 | chr4:94099079 |
| 10 | - | chr5:3143128-21120063 | CEL-5_3149134 | chr5:3143128 |
| 11 | - | chr5:84265109-97939375 | rs13478386 | chr5:90500266 |
